# Characterization of Some Salt-Tolerant Bacterial Hydrolases with Potential Utility in Cultural Heritage Bio-Cleaning

**DOI:** 10.3390/microorganisms10030644

**Published:** 2022-03-17

**Authors:** Robert Ruginescu, Madalin Enache, Octavian Popescu, Ioana Gomoiu, Roxana Cojoc, Costin Batrinescu-Moteau, Gabriel Maria, Maria Dumbravician, Simona Neagu

**Affiliations:** 1 Department of Microbiology, Institute of Biology Bucharest of the Romanian Academy, 296 Splaiul Independentei, 60031 Bucharest, Romania; madalin.enache@ibiol.ro (M.E.); opopescu.ubbcluj@gmail.com (O.P.); ioana.gomoiu@ibiol.ro (I.G.); roxana.cojoc@ibiol.ro (R.C.); costinbatrinescu@yahoo.com (C.B.-M.); gabriel.maria@ibiol.ro (G.M.); simona.neagu@ibiol.ro (S.N.); 2 Molecular Biology Center, Institute of Interdisciplinary Research in Bio-Nano-Sciences, Babes-Bolyai-University, 42 Treboniu Laurian Str., 400271 Cluj-Napoca, Romania; 3 Department of Conservation and Restoration, Faculty of Art History, Bucharest National University of Arts, 19 General Constantin Budișteanu, 010773 Bucharest, Romania; mdumbravician@gmail.com

**Keywords:** halotolerant, halophilic, bacteria, enzymes, hydrolase, esterase, cellulase, protease, bio-cleaning, mural paintings

## Abstract

Salt-tolerant enzymes produced by halophilic and halotolerant microorganisms have been proposed to be used in various applications that involve high saline conditions. Considering their biotechnological significance and the current need for more efficient producers of such catalysts, the present study aimed to evaluate the extracellular proteolytic, esterolytic, cellulolytic and xylanolytic activities of some halotolerant strains, and to characterize their functional parameters. A total of 21 bacterial and fungal strains belonging to the genera *Bacillus*, *Virgibacillus*, *Salinivibrio*, *Salinicoccus*, *Psychrobacter*, *Nocardiopsis*, *Penicillium*, *Aspergillus*, and *Emericellopsis* were assayed by quantitative methods. Among them, the members of the *Bacillus* genus exhibited the highest catalytic activities. The exoenzymes produced by three selected *Bacillus* strains were active over wide ranges of salinity, temperature and pH. Proteases were active at 20–80 °C, pH 6–10, and 0–1 M NaCl, while esterases showed good catalytic activities at 20–80 °C, pH 7.5–10, and 0–4 M NaCl. Cellulases and xylanases were active at 20–80 °C, pH 5–10, and 0–5 M NaCl. Due to such properties, these hydrolases could be used in a newly proposed application, namely to clean aged consolidants and organic deposits accumulated over time from the surfaces of salt-loaded wall paintings.

## 1. Introduction

Enzymes are ubiquitous components of living organisms that catalyze metabolic reactions at rates sufficiently fast to sustain life. Besides their vital biological functions, enzymes have multiple commercial uses in different fields: as industrial catalysts (e.g., conversion of starch to glucose in the food industry), as therapeutic agents (e.g., removal of fibrin clots from bloodstream), as analytic reagents (e.g., detection of glucose in blood) and as manipulative tools (e.g., restriction enzymes used to cut DNA) [1,2]. The global market for enzymes in industrial applications, valued at USD 6.4 billion in 2021 [3], is dominated by hydrolases, especially proteases, amylases, lipases, and cellulases. Microorganisms are the major source of enzymes due to the economic, technical and ethical advantages over the biocatalysts obtained from animals and plants [1,4].

Among the various microbial sources of hydrolytic enzymes, microorganisms that survive in extreme environmental conditions have gained increasing attention in the scientific community over the last decades. Their enzymes, often called extremozymes, perform the same catalytic functions as their mesophilic counterparts, but additionally they operate under severe physicochemical conditions that generally inactivate enzymes produced by non-extremophiles. Due to their robustness, extremozymes have proven to be useful in numerous industrial and environmental applications that require harsh conditions, such as high/low temperatures, acidic/alkaline pH, or high salinity [4,5,6].

A group of extremophilic microorganisms that produce biotechnologically valuable hydrolases is represented by halophiles. They are adapted to survive in highly saline environments and are typically categorized on the basis of their salt requirement/tolerance as extreme halophiles (preferring media with 2.5–5.2 M NaCl), moderate halophiles (optimal growth at 0.5–2.5 M NaCl) or halotolerant microorganisms (non–halophiles that tolerate relatively high salt concentrations, often up to 2.5 M NaCl) [7]. Extracellular enzymes produced by both halophilic and halotolerant microorganisms have evolved to retain structural stability and catalytic activity over a wide range of salinities. These salt-adapted macromolecules, compared to their mesophilic counterparts, show an excess of acidic amino acids at the protein surface and a general decrease in hydrophobic amino acid frequency [8,9]. The major applications in which such extremozymes have been proposed to be used are biofuel production [10], food processing [11] and bioremediation of polluted hypersaline environments [12,13].

In recent years, halophilic microorganisms and their salt-tolerant enzymes have found potential utility in the field of conservation and restoration of cultural heritage, namely as tools to clean mural paintings and other artistic stone works [14,15]. It is well known that, under certain environmental conditions, hygroscopic salts present in porous building materials may concentrate and crystallize at the surface of the walls/stone artworks [16,17], thereby limiting bio-cleaning processes based on mesophilic microbial cells or on conventional enzymes [15,18]. Under such conditions, a system based on microorganisms or biocatalysts capable of acting over a wide range of salinities could represent the optimal solution to remove unwanted inorganic (e.g., nitrates, sulfates) or organic compounds (e.g., protein deposits, oils, waxes) from the surfaces of wall paintings and historical monuments. To our knowledge, there is only one study that reports the use of a halophilic bacterial strain (i.e., *Halomonas campaniensis*) in bio-cleaning treatments [14]. In their study, the authors have achieved the removal of nitrate efflorescence from the surfaces of stone samples. However, the halophilic strain was selected for its ability to reduce nitrates and not for its particular potential to be used under saline conditions [14].

Several studies have successfully reported the use of both mesophilic bacteria (particularly the *Pseudomonas* and *Desulfovibrio* strains) and commercially available enzymes for the restoration of various artworks, such as frescoes, wall paintings and stone sculptures [19,20,21,22,23]. These biological methods are more advantageous (i.e., less aggressive, more specific, and more sustainable) compared with the traditional chemical and mechanical techniques. Based on these considerations, we have recently proposed the use of a system based on salt-tolerant hydrolases entrapped in a gel matrix (e.g., Agarart, Kelcogel, Vanzan) to remove organic residues (e.g., glue, casein, resins used in previous restorative interventions) and deposits of atmospheric contaminants (e.g., hydrocarbons, lipids, proteins) from salt-loaded wall paintings [15]. Considering that these compounds may serve as nutrients for heterotrophic microorganisms, their removal is crucial to avoid a future microbial colonization and the subsequent deterioration of cultural artifacts [24].

Despite the great potential utility of salt-tolerant enzymes in various biotechnological applications, they are not currently produced at an industrial scale, and therefore they are not commercially available. In this context, in the present study, we aimed to (1) quantitatively evaluate the extracellular proteolytic, esterolytic, cellulolytic, and xylanolytic activities of different halotolerant bacteria and microfungi, (2) select the strains with high enzyme activities, (3) characterize the catalytic activities of concentrated enzymes in different physico-chemical conditions, and (4) test the possibility of using halotolerant esterase in the field of bio-cleaning of mural paintings by qualitatively evaluating their ability to remove acrylic resin Paraloid B-72 and oil from painted laboratory models. Consequently, the present research provides novel efficient sources of salt-tolerant enzymes that could be used in various applications where saline conditions inhibit enzymatic transformations, such as the cleaning of wall paintings affected by efflorescence.

## 2. Materials and Methods

### 2.1. Bacterial and Fungal Sources of Hydrolytic Enzymes

Bacterial and fungal strains used in the present study were isolated previously from different brackish, saline or hypersaline lakes located in Romania [25]. Their ability to produce extracellular hydrolases was also qualitatively determined in our previous work [25]. For the current investigation, a total of 21 strains with promising proteolytic, esterolytic, cellulolytic or xylanolytic activities were selected from our laboratory collection (Table S1). They were represented by 16 bacterial strains belonging to the genera *Bacillus*, *Virgibacillus*, *Salinivibrio*, *Salinicoccus*, *Psychrobacter* and *Nocardiopsis*, and five fungal strains belonging to the genera *Penicillium*, *Aspergillus* and *Emericellopsis*. All the strains were previously categorized as halotolerant (growth in media with 0–2 M NaCl) or as extremely halotolerant (growth at 0–3 M NaCl) [25], thereby being potential sources of salt-tolerant exoenzymes.

### 2.2. Taxonomic Identification of the Fungal Strains

The fungal strains which were not taxonomically characterized in our previous study [25] were identified by PCR amplification and sequencing of the ITS1-5.8S-ITS2 gene. To this end, genomic DNA was extracted from mycelia using the Quick-DNA Fungal/Bacterial Kit (Zymo Research, Irvine, CA, USA) following the manufacturer’s protocol. PCR amplification of the ITS gene was carried out in a 50 μL final reaction volume containing 1× DreamTaq Green Master Mix (Thermo Fisher Scientific, Waltham, MA, USA), 0.2 μM of each primer (ITS1 and ITS4) [26], 20–50 ng DNA template and nuclease-free water. PCR reactions were performed under the following conditions: 3 min denaturation at 95 °C, 35 cycles of 30 s denaturation at 95 °C, 30 s annealing at 53 °C, 1 min extension at 72 °C, and a final extension step of 7 min at 72 °C. Amplicons were purified using the DNA Clean & Concentrator Kit (Zymo Research, Irvine, CA, USA) and sequenced by a commercial sequencing service provider (Macrogen Europe, Amsterdam, The Netherlands). Sequencing inaccuracies were edited using the CodonCode Aligner software and the resulting sequences were compared against the NCBI database using BLASTN [27]. The partial ITS sequences were deposited in GenBank under the accession numbers OL454638–OL454642 (Table S1).

### 2.3. Culture Conditions for Enzyme Production

Bacterial and fungal strains were routinely cultured on HM growth medium [28] containing 0.5 M NaCl (pH 7.2) at 30 °C. For the production of enzymes (i.e., protease, esterase, cellulase and xylanase), the HM medium was modified to include one of the following substrates (g⋅L^−1^): casein (10), Tween-80 (10), carboxymethyl cellulose (CMC) (5), or xylan (10). Glucose and proteose-peptone were excluded from their composition. These media were inoculated with 1% (*v*/*v*) of a pre-culture grown in HM medium for 24 h (in the case of bacterial strains) or for three days (in the case of fungal strains). Incubation was performed at 30 °C for 72 h (in the case of bacteria) or for five days (in the case of fungi), under continuous agitation (150 rpm). Following the incubation period, the cells were harvested by centrifugation at 7500× *g* for 20 min and the cell-free supernatant was assayed for proteolytic, esterolytic, cellulolytic or xylanolytic activities.

### 2.4. Quantitative Evaluation of Hydrolytic Activities

#### 2.4.1. Protease Activity Assay

The proteolytic activity was assayed using the method of Anson [29] with some modifications [30]. Briefly, 1 mL of suitably diluted sample was added to 5 mL of casein solution (5 mg⋅mL^−1^) in 50 mM potassium phosphate buffer (pH 8). This assay mixture was incubated at 37 °C for exactly 10 min (in the case of concentrated enzymes) or 20 min (in the case of cell–free supernatants), and the reaction was stopped by adding 5 mL of 0.11 M trichloroacetic acid (TCA). The mixture was incubated at 37 °C for 30 min and then was filtered through Whatman #1 filter paper to remove precipitates. To 2 mL of the filtrate were added 5 mL of 0.5 M sodium carbonate solution and 1 mL of Folin–Ciocalteu reagent (1:2 diluted with distilled water). After an incubation of 30 min at 37 °C, the absorbance was measured at 660 nm. A blank control was also prepared by adding TCA before the enzyme.

The amount of tyrosine released from casein was estimated based on a standard curve of tyrosine generated in the range from 1 to 200 μg. The proteolytic activity was expressed in units per milliliter (U⋅mL^−1^) or in units per milligram of protein (U⋅mg^−1^). One unit of protease activity was defined as the amount of enzyme that releases 1 μg of tyrosine per minute under the assay conditions. All the experiments were performed in triplicate.

#### 2.4.2. Esterase Activity Assay

The esterase activity was determined by measuring the amount of *p*-nitrophenol (*p*-NP) released from *p*-nitrophenyl butyrate (*p*-NPB) [31]. Briefly, 250 μL of suitably diluted sample was added to 730 μL of 50 mM potassium phosphate buffer (pH 7.5), and the mixture was pre-incubated at 37 °C for 5 min. Then, 20 μL of 50 mM *p*-NPB (dissolved in absolute ethanol) was added, and the reaction mixture was incubated at 37 °C for exactly 10 min. At the end of the incubation, the reaction tubes were placed on ice and the absorbance of *p*-NP was measured immediately at 410 nm. A blank control was prepared in the same manner except that the enzyme was added in the reaction mixture immediately before reading the absorbance.

The amount of *p*-NP released from the substrate was determined using the Beer-Lambert equation, knowing that the molar extinction coefficient of *p*-NP was determined as 10,400 M^−1^⋅cm^−1^ [32]. One unit of esterase activity was defined as the amount of enzyme required to release 1 μmol of *p*-NP per minute under the assay conditions. All tests were performed in triplicate.

#### 2.4.3. Cellulase Activity Assay

The cellulase activity was measured using the 3,5-dinitrosalicylic acid (DNS) reducing sugar method [33] with CMC as substrate. The reaction was initiated by adding 1 mL of suitably diluted sample to 1 mL of 1% (*w*/*v*) CMC in 50 mM potassium phosphate buffer (pH 7), and incubating the tubes at 37 °C. The reaction was terminated after 30 min by adding 3 mL of DNS reagent. The tubes were incubated for 15 min in a boiling water bath. Then, 1 mL of 40% (*w*/*v*) potassium sodium tartrate solution was added immediately and the tubes were cooled at room temperature over 20 min. The absorbance of the reaction solutions was measured at 540 nm. An enzyme blank was prepared by adding the DNS reagent before the enzyme solution.

The amount of glucose released from the substrate was estimated based on a standard curve of glucose generated in the range 1 to 7.5 μmol. One unit of cellulase activity was defined as the amount of enzyme required to release 1 μmol of glucose per minute under the assay conditions. All tests were performed in triplicate.

#### 2.4.4. Xylanase Activity Assay

The xylanase activity assay was based on the DNS reducing sugar method described above [33], with the only exception that the substrate was represented by 1% (*w*/*v*) xylan in 50 mM potassium phosphate buffer (pH 7).

The amount of xylose released from the substrate was estimated based on a standard curve of xylose generated in the range 0.5 to 4 μmol. One unit of xylanase activity was defined as the amount of enzyme required to release 1 μmol of xylose per minute under the assay conditions. All tests were performed in triplicate.

### 2.5. Precipitation of Extracellular Hydrolases

The exoenzymes found in the culture broth were concentrated by acetone precipitation [34]. To this end, one volume of cell-free supernatant was added to four volumes of cold acetone, and the mixture was kept overnight at 4 °C. Proteins were pelleted by centrifugation at 10,000× *g* for 30 min (at 4 °C). The protein pellets were air dried for 30 min at room temperature and then were resuspended in a minimal volume of 50 mM potassium phosphate buffer, pH 7.0 (in the case of cellulases and xylanases), pH 7.5 (in the case of esterases) or pH 8.0 (in the case of proteases).

Protein concentrations in the cell-free supernatants and crude precipitates were estimated by Lowry’s method [35] using bovine serum albumin as the standard.

### 2.6. Evaluation of the Effects of Physicochemical Factors on the Activity and Stability of Enzymes

The effects of temperature, pH and salts on the activity of concentrated enzyme solutions were determined by performing the assays as described in Section 2.4, with the exceptions that reactions were conducted at different temperatures (20–80 °C), pH values (4–10) and NaCl concentrations (0–5 M). The following buffers were used to generate different pH conditions: 0.1 M phosphate–citrate (pH 4.0 and 5.0), 0.1 M potassium phosphate (pH 6.0 and 7.0), 0.1 M Tris–HCl (pH 8.0 and 9.0), and 0.1 M glycine-NaOH (pH 10).

The thermal stability of enzymes was determined by incubating aliquots of each enzyme at different temperatures (4, 20, 40, 60 and 80 °C) for 48 h. The catalytic activities were generally assayed at 24 h intervals as described in Section 2.4.

### 2.7. Preliminary Evaluation of the Efficacy of Halotolerant Esterases in Bio-Cleaning Applications

**Figure 1:**
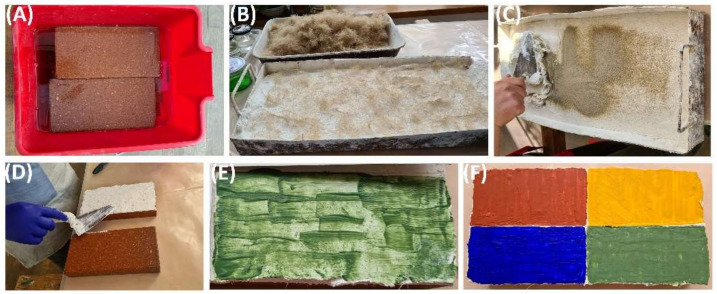
Preparation of the painted laboratory models. The stages of the procedure (**A**–**F**) were described in the main text.

The efficacy of utilizing halotolerant esterases produced by *Bacillus* sp. BA N P3.3 in the cleaning of wall paintings was qualitatively evaluated on laboratory models represented by painted bricks that simulated the mural surface. Briefly, the following steps were performed to obtain the laboratory models: (a) wetting the bricks, (b) inserting the lime and the tow in special trays, (c) incorporating the sand, (d) placing the mortar on the wet bricks, (e) applying the pigments (red, yellow, blue, green), and (f) slow drying for 30 days (Figure 1). Then, three layers of acrylic resin Paraloid B-72 or sunflower oil were applied on the painted surfaces using a brush.

The cleaning treatments involved the application of the concentrated esterase solution obtained by acetone precipitation on the surface of the painted laboratory models (1 mL enzyme/6.25 cm^2^) and their incubation for 10 h at room temperature. Then, the enzyme extract was removed from the treated surfaces using cotton swabs moistened in distilled water. Morphological changes due to the hydrolysis of Paraloid B-72 or sunflower oil were examined by optical and scanning electron microscopy (SEM). Negative controls were treated in the same manner as the samples, with the only exception that the enzyme solution was replaced by the enzyme buffer.

## 3. Results

### 3.1. Characterization of Protease Activities

**Figure 2:**
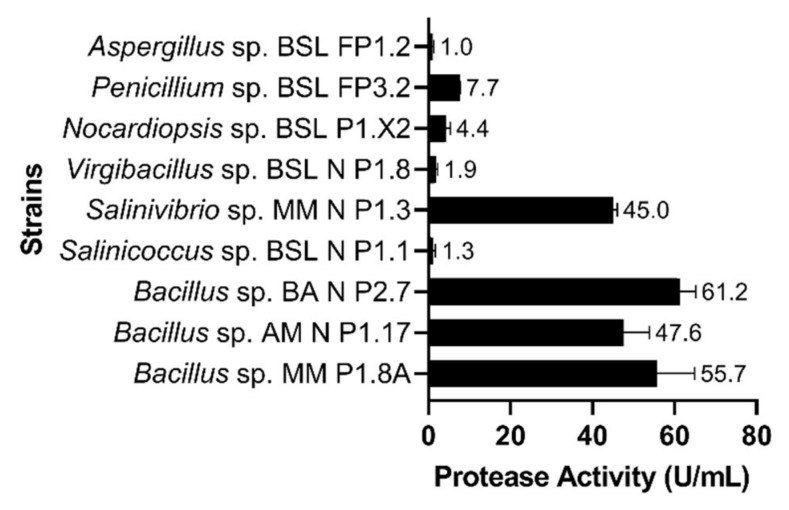
Protease activities, expressed in units per mL, in the culture supernatant. The results are presented as averages of three replicates, and error bars represent the standard deviation of the mean.

Among the nine microbial strains tested, the highest proteolytic activities were detected in the culture supernatant of *Bacillus* sp. BA N P2.7 (61 U⋅mL^−1^), *Bacillus* sp. MM P1.8A (55 U⋅mL^−1^), *Bacillus* sp. AM N P1.17 (47 U⋅mL^−1^) and *Salinivibrio* sp. MM N P1.3 (45 U⋅mL^−1^) (Figure 2).

For instance, *Bacillus* sp. BA N P2.7 synthesized, in a relatively short period of time (i.e., 72 h), the amount of protease required to release a quantity of tyrosine 48 times higher than that generated by the enzymes produced by *Salinicoccus* sp. BSL N P1.1. The other bacterial and fungal strains tested showed relatively low proteolytic activities, between 1 and 7 U⋅mL^−1^ (Figure 2). Based on the high proteolytic activity and other physiological advantages (i.e., the rapid growth rate, the ability to grow over a wide range of salinities, and to produce multiple exoenzymes), *Bacillus* sp. AM N P1.17 was selected for subsequent experiments. This strain, previously isolated from a brackish lake [25], showed 100% homology of the 16S rRNA gene sequence with that of *Bacillus inaquosorum* BGSC 3A28. It was able to grow over a wide range of temperatures (15–50 °C), pH values (5–9) and salinities (0–12%, *w*/*v*), while its optimal conditions for growth were at 40 °C, pH 7–8, and 0–5% (*w*/*v*) salts (Figure S1). Additionally, as previously described [25], *Bacillus* sp. AM N P1.17 was able to produce six types of extracellular hydrolases, namely proteases, esterases, amylases, cellulases, xylanases, and pectinases.

Under standard assay conditions (i.e., 37 °C, pH 8), the specific protease activity of the culture supernatant was 1.75 U⋅mg^−1^. After concentrating proteins by acetone precipitation, the proteolytic activity was enhanced to 11.6 U⋅mg^−1^, with a yield of about 46% (Table 1).

**Figure 3:**
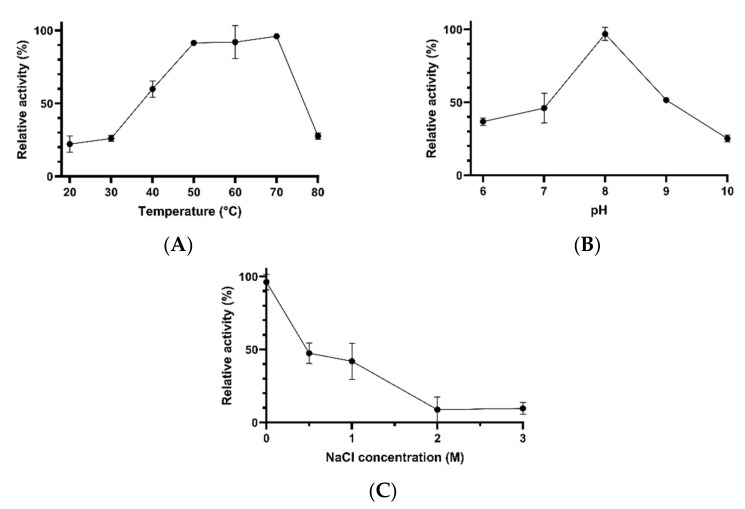
The effects of temperature (**A**), pH (**B**) and NaCl concentration (**C**) on the activity of concentrated proteases obtained from *Bacillus* sp. AM N P1.17. Error bars represent the standard deviation of the mean (*n* = 3).

Concentrated proteases were active over a wide range of temperatures (20–80 °C) and pH values (6–10). The highest protease activity was detected at temperatures between 50 and 70 °C. At 20–30 °C, the enzymes exhibited about 22–26% of the maximal activity. Similarly, at 80 °C, the enzymes exhibited about 27% of their optimal activity (Figure 3A). The pH had also an important effect on proteolytic activity. While the optimal activity was detected at pH 8, the other pH values tested (6, 7, 9, and 10) significantly inhibited the catalytic activity. At pH 6 and 7, the enzymes retained about 37% and 46% of the maximal activity, respectively. Moreover, at pH 9 and 10, the proteases showed about 51% and 25% of the optimal activity, respectively (Figure 3B). The enzymes exhibited optimal activity in the absence of salts. However, they were able to retain about 47% and 42% of their maximal activity at 0.5 M NaCl and at 1 M NaCl, respectively. At higher salt concentrations, the protease activity was almost completely inhibited (Figure 3C).

Proteases retained 88%, 77%, and 15% of the maximal activity after 24 h of incubation at 4 °C, 20 °C, and 40 °C, respectively. After 48 h at the same temperatures, proteases retained less than half of the activity determined after 24 h of incubation. At higher temperatures, enzymes lost their catalytic activity completely (Figure S2A).

### 3.2. Characterization of Esterase Activities

**Figure 4:**
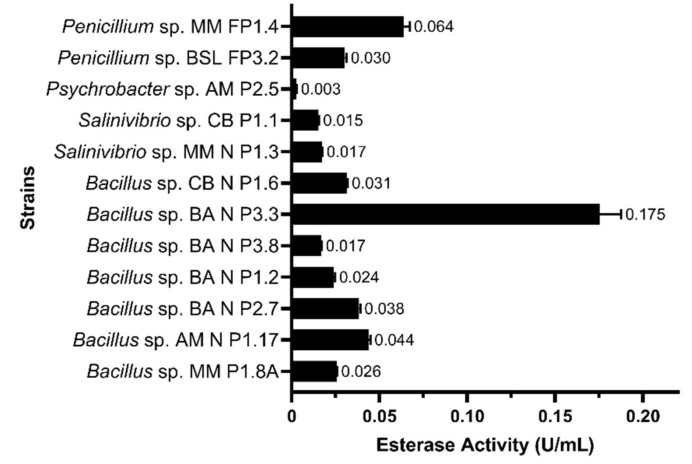
Esterase activities, expressed in units per mL, in the culture supernatant. The results are presented as averages of three replicates, and error bars represent the standard deviation of the mean.

The highest esterolytic activities, among the twelve microbial strains tested, were produced by *Bacillus* sp. BA N P3.3 (0.175 U⋅mL^−1^), followed by *Penicillium* sp. MM FP1.4 (0.064 U⋅mL^−1^). Other *Bacillus* strains (i.e., AM N P1.17, BA N P2.7, and CB N P1.6) showed moderate esterolytic activities (0.031–0.044 U/mL), while the lowest catalytic activity was detected in the culture supernatant of *Psychrobacter* sp. AM P2.5 (0.003 U/mL) (Figure 4).

*Bacillus* sp. BA N P3.3 was selected for subsequent experiments based on both the relatively high ability to produce esterases and the faster growth rate compared with *Penicillium* sp. MM FP1.4. This strain showed 100% homology of the partial 16S rRNA gene sequence with *Bacillus zhangzhouensis* MCCC 1A08372 and *Bacillus pumilus* ATCC 7061. It grew at different temperatures (15–40 °C), pH values (5–10) and salt concentrations (0–12%, *w*/*v*), while its optimal growth conditions were at 30 °C, pH 7–8, and 0–5% (*w*/*v*) salts (Figure S3). Moreover, *Bacillus* sp. BA N P3.3 was able to produce extracellular proteases, esterases, cellulases and xylanases.

The specific esterase activity of the culture supernatant was 0.016 U⋅mg^−1^ under standard assay conditions (i.e., 37 °C, pH 7.5). After concentrating proteins by acetone precipitation, the esterolytic activity was enhanced to 0.124 U⋅mg^−1^ (Table 2).

**Figure 5:**
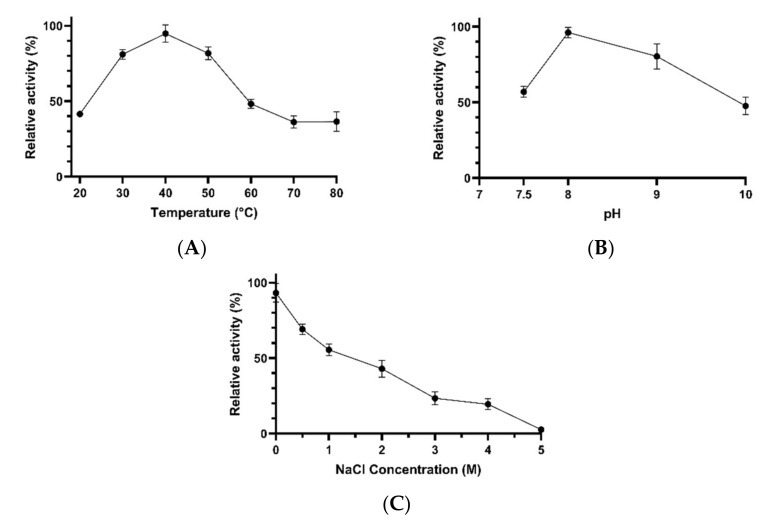
The effects of temperature (**A**), pH (**B**) and NaCl concentration (**C**) on the activity of concentrated esterases obtained from *Bacillus* sp. BA N P3.3. Error bars represent the standard deviation of the mean (*n* = 3).

Concentrated esterases were active over a wide range of temperatures (20–80 °C) and salt concentrations (0–4 M NaCl). The highest esterase activity was detected at 40 °C, while at 30 °C and 50 °C, the enzymes showed about 81% of the maximal activity. Lower catalytic activities were detected at 20 °C (41% of the maximal activity), 60 °C (48% of the maximal activity), and 70–80 °C (36% of the maximal activity) (Figure 5A). The pH at which the enzymes showed optimal activity was 8. At pH 9 and 10, the esterolytic activity was reduced to about 80% and 47% of the maximal value, respectively (Figure 5B). Moreover, as shown in Figure 5C, the esterolytic activity decreased with increasing salt concentration. While the optimal activity was detected in the absence of salts, the esterases exhibited about 55%, 43%, 23%, and 19% of the maximal activity at 1, 2, 3, and 4 M NaCl, respectively. The activity was almost completely absent at 5 M NaCl.

Esterases were stable at temperatures between 4 and 40 °C for at least 48 h. At higher temperatures, the enzymes lost their activity after 2 h of incubation (Figure S2B).

### 3.3. Characterization of Cellulase Activities

**Figure 6:**
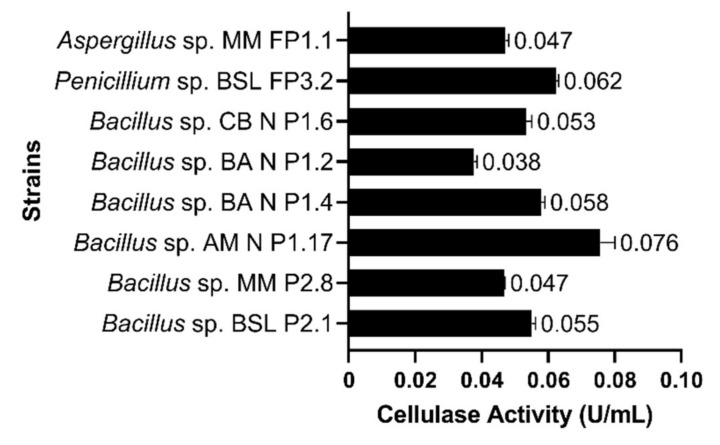
Cellulase activities, expressed in units per mL, in the culture supernatant. The results are presented as averages of three replicates; error bars represent the standard deviation of the mean.

Six bacterial strains belonging to the genus *Bacillus* and two fungal strains belonging to *Penicillium* and *Aspergillus* genera were quantitatively assayed for extracellular cellulolytic activities. Among them, *Bacillus* sp. AM N P1.17 produced the highest cellulase activity (0.076 U⋅mL^−1^), followed by *Penicillium* sp. BSL FP3.2 (0.062 U⋅mL^−1^) (Figure 6). Therefore, *Bacillus* sp. AM N P1.17 was selected for subsequent experiments.

The specific cellulase activity of the culture supernatant was 0.008 U⋅mg^−1^ under standard assay conditions (i.e., 37 °C, pH 7). After concentrating proteins by acetone precipitation, the cellulolytic activity was enhanced to 0.032 U⋅mg^−1^ (Table 3).

**Figure 7:**
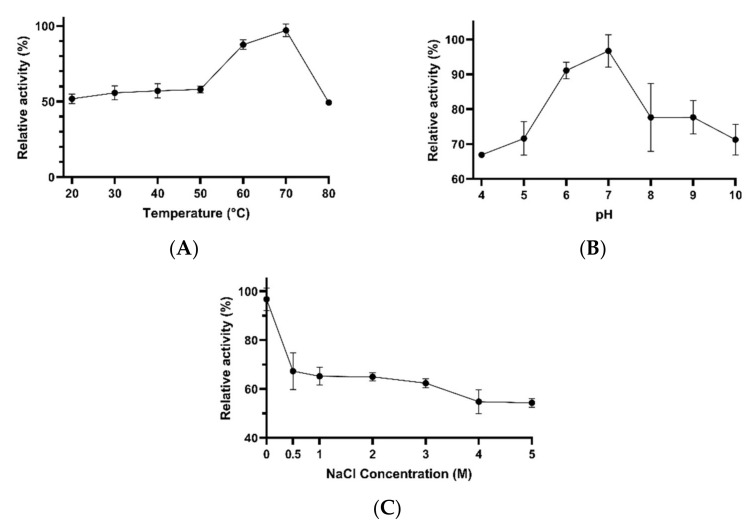
The effects of temperature (**A**), pH (**B**) and NaCl concentration (**C**) on the activity of concentrated cellulases obtained from *Bacillus* sp. AM N P1.17 Error bars represent the standard deviation of the mean (*n* = 3).

Concentrated cellulases were active over a wide range of temperatures (20–80 °C), pH values (4–10) and salt concentrations (0–5 M NaCl). The highest cellulase activity was obtained at 70 °C, and about 87% of the maximal activity was detected at 60 °C. At the other tested temperatures, the enzymes showed 49–58% of the maximal activity (Figure 7A). Cellulases exhibited optimal activity at pH 7, and about 91% of the maximal activity was observed at pH 6 (Figure 7B). The catalytic activity decreased with increasing NaCl concentration. About 65% of the optimal activity was detected when the reaction mixture contained 1–3 M NaCl. At higher salt concentrations (i.e., 4–5 M NaCl), the cellulase activity decreased to about 54% of the maximal value detected (Figure 7C).

Cellulases were stable at temperatures between 4 and 40 °C for at least 48 h. At 60 °C, cellulases retained 73% of the maximal activity after 2 h of incubation, but they completely lost their activity after 24 h of incubation (Figure S2C).

### 3.4. Characterization of Xylanase Activities

**Figure 8:**
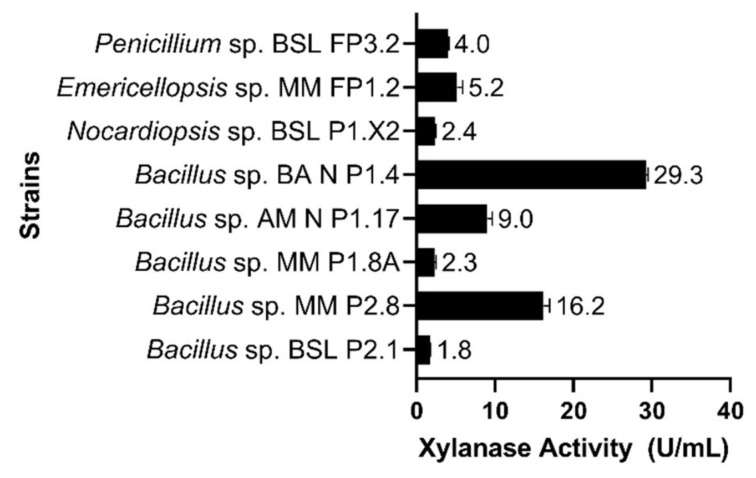
Xylanase activities, expressed in units per mL, in the culture supernatant. The results are presented as averages of three replicates; error bars represent the standard deviation of the mean.

Among the eight microbial strains tested, *Bacillus* sp. BA N P1.4 exhibited the highest xylanolytic activity (29.3 U⋅mL^−1^), followed by *Bacillus* sp. MM P2.8 (16.2 U⋅mL^−1^). The other bacterial and fungal strains tested showed much lower xylanase activities, between 1.8 and 9 U⋅mL^−1^ (Figure 8).

Considering its high xylanolytic activity, *Bacillus* sp. BA N P1.4 was selected for subsequent experiments. Based on the 16S rRNA gene sequence analysis, this strain was closely related to *Bacillus zhangzhouensis* MCCC 1A08372 (100% homology). Furthermore, it showed a high similarity with the esterase-producing strain *Bacillus* sp. BA N P3.3. It grew at 15–50 °C (optimum 30 °C), at pH 5–10 (optimum 7–8), and at a total salt concentration between 0 and 12% (*w*/*v*), with an optimum at 0–5% (*w*/*v*) (Figure S4). As previously described [25], in addition to xylanases, *Bacillus* sp. BA N P1.4 had the ability to produce proteases, esterases, and cellulases.

Under standard assay conditions (i.e., 37 °C, pH 7), the specific xylanase activity of the culture supernatant was 2.43 U⋅mg^−1^. After concentrating proteins by acetone precipitation, the xylanolytic activity was enhanced to 11.3 U⋅mg^−1^, with a yield of about 72% (Table 4).

**Figure 9:**
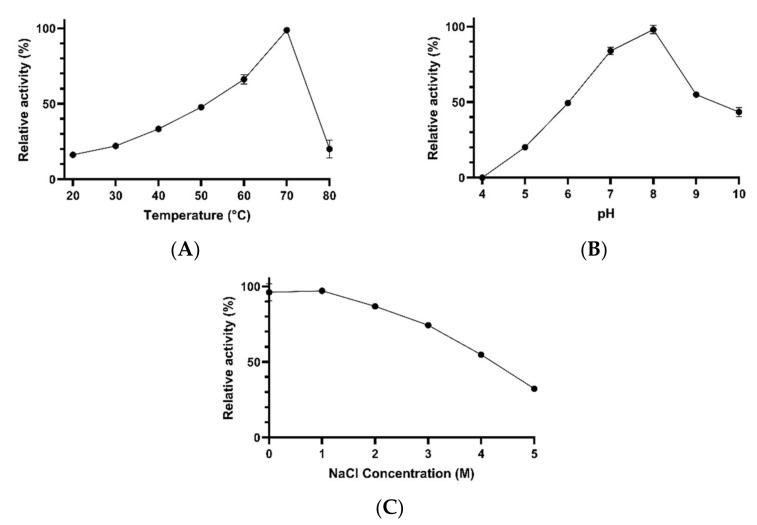
The effects of temperature (**A**), pH (**B**) and NaCl concentration (**C**) on the activity of concentrated xylanases obtained from *Bacillus* sp. BA N P1.4 Error bars represent the standard deviation of the mean (*n* = 3).

Concentrated xylanases were active at different temperatures (20–80 °C), pH values (5–10), and salt concentrations (0–5 M NaCl). The xylanolytic activity increased gradually with increasing temperature from 20 to 70 °C, at which point the highest activity was achieved. At 80 °C, the catalytic activity dropped sharply to about 20% of the activity recorded at 70 °C. At 20, 30, 40, 50, and 60 °C, concentrated xylanases showed about 16%, 22%, 33%, 47%, and 66% of the maximal activity, respectively (Figure 9A). The pH also had a significant effect on xylanolytic activity. In this regard, the activity increased gradually from pH 5 to the optimal value, i.e., pH 8. At pH 9 and 10, the activity decreased to about 55% and 43% of the maximal activity, respectively. At pH 5, 6, and 7, xylanases exhibited 20%, 49%, and 83% of the activity recorded at pH 8, respectively. No activity was detected at pH 4 (Figure 9B). The xylanase activity decreased with increasing salt concentration from 0 M to 5 M NaCl. At 1 M NaCl, the activity was about 97% of that recorded at 0 M NaCl, and gradually dropped to about 32% (at 5 M NaCl) (Figure 9C).

Xylanases retained 60–100% of the maximal activity after 42 h of incubation at temperatures between 4 and 40 °C. At higher temperatures, the enzymes lost their activity completely (Figure S2D).

### 3.5. The Efficacy of Halotolerant Esterases in the Removal of Paraloid B-72 and Oil

**Figure 10:**
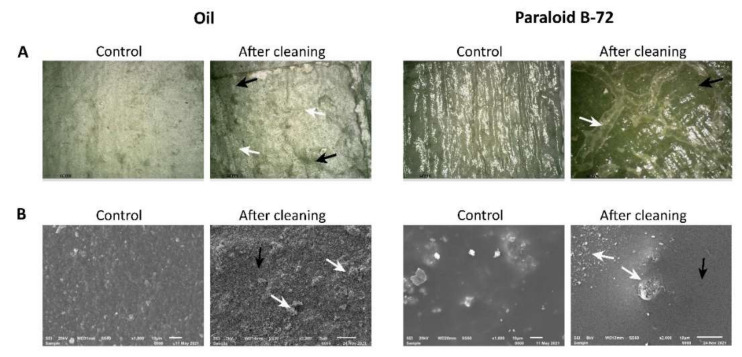
The efficacy of esterase-based cleaning treatments on murals (painted laboratory models) covered by sunflower oil or Paraloid B-72, evaluated by optical microscopy (**A**) and SEM (**B**). The white arrows indicate the cleaned areas, while the black arrows indicate the areas where the targeted substrates were not removed due to the uneven distribution of the enzyme on samples’ surfaces.

The esterase-treated surfaces of the painted laboratory models showed morphological changes that indicated the partial removal of both acrylic resin Paraloid B-72 and sunflower oil. In comparison to the untreated control, the bio-cleaned surfaces appeared to be brighter and less reflective (Figure 10A). Moreover, SEM analyses showed textural differences between treated and untreated areas. In this regard, the Paraloid B-72 layer appeared to be discontinuously hydrolyzed (Figure 10B). The partial removal of targeted substrates could be attributed to the uneven distribution of enzymes on the treated surfaces. This phenomenon could be prevented by entrapping the enzymes in a gel matrix, but this hypothesis must be tested and confirmed in further experiments.

In the case of oil-enriched laboratory models, the efficacy of bio-cleaning treatments could not be correctly evaluated on red and yellow pigmented areas due to the detachment of the painted layers after the application of the enzyme (Figure S5). However, samples enriched with Paraloid B-72, which served as a consolidant, were not affected by this phenomenon. In this case, it was observed that the efficacy of the bio-cleaning treatments was not significantly affected by the type of pigment.

## 4. Discussion

Salt-tolerant enzymes have been proposed to be used in various applications involving highly saline conditions, such as biofuel production and bioremediation processes [10,11,12,13]. Numerous halophilic/halotolerant microbial species capable of producing different extracellular hydrolases (e.g., amylase, cellulase, xylanase, inulinase, pectinase, protease, and lipase) have been isolated and identified over recent decades [25,36,37,38,39,40]. Several of these macromolecules have been purified and characterized, thereby unveiling multiple biotechnologically valuable properties. For instance, in addition to salt tolerance, some enzymes synthesized by halophilic/halotolerant microorganisms have shown to be active and stable under extreme conditions of pH and temperature, and in the presence of metal ions, organic solvents, and detergents [10,41,42,43,44]. However, to the best of our knowledge, these robust biocatalysts are not produced at industrial scales, and current studies are focused mostly on improving their production yields [45,46,47].

Considering the current need for more efficient producers of salt-tolerant enzymes, the present study reported the discovery of novel halotolerant strains capable of producing relatively high quantities of proteases, esterases, cellulases, and xylanases. Among the 21 microbial strains tested, the members of the *Bacillus* genus exhibited the highest catalytic activities. This observation is in agreement with numerous previous studies that have highlighted the remarkable potential of *Bacillus* species to produce various bioactive molecules [48,49].

The exoenzymes produced by three selected strains (i.e., *Bacillus* sp. AM N P1.17, BA N P3.3, and BA N P1.4) were active over a wide range of salt concentrations, temperatures and pH values. Due to such functional properties, these hydrolases could be suitable for a newly proposed biotechnological application [15], namely as tools to clean organic consolidants/deposits from the surfaces of salt-loaded wall paintings. Considering that the four characterized hydrolases presented optimal catalytic activities at relatively high temperatures (i.e., 40–70 °C), their utilization in bio-cleaning applications could be advantageous, particularly for outdoor murals in the summer season. However, the enzymes were also active at 20 °C and could therefore be effective in processes performed indoors, but in this case longer exposure times could be required.

The ability of halotolerant esterase produced by *Bacillus* sp. BA N P3.3 to remove a widely utilized consolidant, i.e., Paraloid B-72, and oil from the painted surfaces of laboratory models represented a promising result in the field of bio-cleaning of mural paintings, stone artworks, as well as icons. To the best of our knowledge, this is the first report that utilized a salt-tolerant enzyme in a bio-cleaning process. Similarly, proteases could be used to clean adhesives and other consolidating materials (e.g., casein, calcium caseinate) that have been applied, but not completely removed, during previous restorations. Over time, these compounds may undergo alterations, thereby causing aesthetic damage to the wall paintings [19]. Cellulases and xylanases may be useful in the removal of lignocellulosic materials that have been used in the reinforcement of the mortar, but which sometimes have not been completely covered by it (due to technical errors), causing unaesthetic effects at the painting surface. In addition, considering that such organic residues/contaminants may serve as nutrients for both bacteria and fungi, their removal is essential to avoid microbial colonization and the subsequent deterioration of the wall paintings.

Further studies must be performed to determine if enzymes entrapped in different gel matrices will produce a more uniform cleaning. Subsequently, the efficacy of these systems should also be tested by other advanced methods (e.g., colorimetry, Fourier transform infrared spectroscopy) on real artworks affected by efflorescence. However, the production cost of enzymes could represent a major drawback for the widespread use of enzyme-based bio-cleaning. In this context, the use of concentrated crude enzymes instead of highly purified ones could represent an adequate solution to streamline the process.

## Figures and Tables

**Table 1:** Summary of extracellular protease precipitation.

Sample	Total Activity (U)	Total Protein (mg)	Specific Activity (U⋅mg^−1^)	Yield (%)	Purification Fold
Culture supernatant	47.5	27.1	1.75	100	1
Precipitated proteins	22	1.9	11.6	46.3	6.6

**Table 2:** Summary of extracellular esterase precipitation.

Sample	Total Activity (U)	Total Protein (mg)	Specific Activity (U⋅mg^−1^)	Yield (%)	Purification Fold
Culture supernatant	0.175	10.75	0.016	100	1
Precipitated proteins	0.06	0.485	0.124	34	7.75

**Table 3:** Summary of extracellular cellulase precipitation.

Sample	Total Activity (U)	Total Protein (mg)	Specific Activity (U⋅mg^−1^)	Yield (%)	Purification Fold
Culture supernatant	0.071	8.66	0.008	100	1
Precipitated proteins	0.043	1.33	0.032	60.5	4

**Table 4:** Summary of extracellular xylanase precipitation.

Sample	Total Activity (U)	Total Protein (mg)	Specific Activity (U⋅mg^−1^)	Yield (%)	Purification Fold
Culture supernatant	29.2	12	2.43	100	1
Precipitated proteins	21	1.86	11.3	72	4.65

## Supplementary Materials

The following supporting information can be downloaded at: https://www.mdpi.com/article/10.3390/microorganisms10030644/s1, Table S1: The bacterial and fungal strains used in the present study and the accession numbers of their 16S rRNA/ITS gene sequence, Figure S1: The effect of temperature (A), pH (B) and salt concentration (C) on the growth of *Bacillus* sp. AM N P1.17, Figure S2. The effect of temperature on the stability of proteases (A), esterases (B), cellulases (C) and xylanases (D) produced by the selected strains of *Bacillus* sp., Figure S3: The effect of temperature (A), pH (B) and salt concentration (C) on the growth of *Bacillus* sp. BA N P3.3, Figure S4: The effect of temperature (A), pH (B) and salt concentration (C) on the growth of *Bacillus* sp. BA N P1.4, Figure S5: Oil-enriched laboratory model after the treatment with esterase.
