# Supplementary material for: Characterization of Some Salt-Tolerant Bacterial Hydrolases with Potential Utility in Cultural Heritage Bio-Cleaning
Source: Microorganisms. 2022 Mar 17;10(3):644. doi: 10.3390/microorganisms10030644 (PMC8949325; doi:10.3390/microorganisms10030644)
Supplement: Supplementary file 1 [file microorganisms-10-00644-s001.zip › microorganisms-1611910-supplementary.pdf]

Table S1. The bacterial and fungal strains used in the present study and the accession numbers of their 16S rRNA/ITS gene sequence.

Genus	Strain	GenBank accession no.
Bacillus sp.	MM P1.8A	MW036380
Bacillus sp.	AM N P1.17	MW036408
Bacillus sp.	BA N P2.7	MW036426
Bacillus sp.	BA N P1.2	MW036436
Bacillus sp.	BA N P3.3	OL454645
Bacillus sp.	BA N P3.8	MW036437
Bacillus sp.	CB N P1.6	MW036433
Bacillus sp.	BSL P2.1	MW036388
Bacillus sp.	MM P2.8	MW036378
Bacillus sp.	BA N P1.4	MW036427
Virgibacillus sp.	BSL N P1.8	MW036383
Salinivibrio sp.	CB P1.1	MW036411
Salinivibrio sp.	MM N P1.3	MW036396
Salinicoccus sp.	BSL N P1.1	MW036374
Psychrobacter sp.	AM P2.5	MW036416
Nocardiopsis sp.	BSL P1.X2	MW036395
Penicillium sp.	BSL FP3.2	OL454640
Penicillium sp.	MM FP1.4	OL454639
Aspergillus sp.	BSL FP1.2	OL454642
Aspergillus sp.	MM FP1.1	OL454641
Emericellopsis sp.	MM FP1.2	OL454638

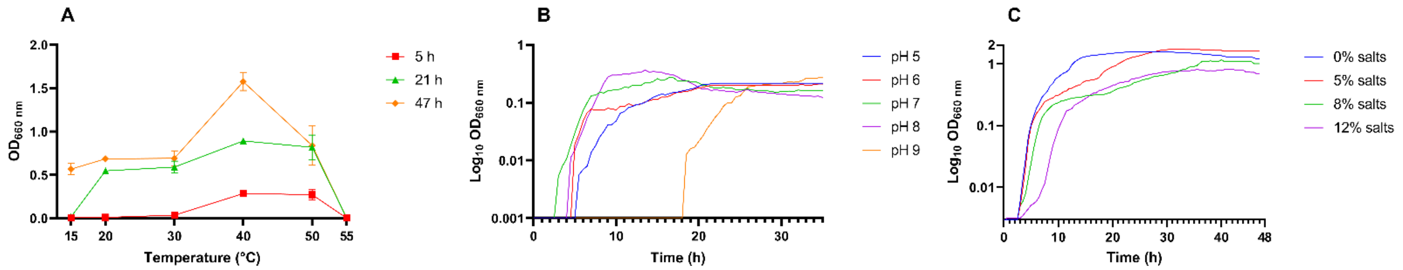

Figure S1. The effect of temperature (**A**), pH (**B**) and salt concentration (**C**) on the growth of *Bacillus* sp. AM N P1.17.

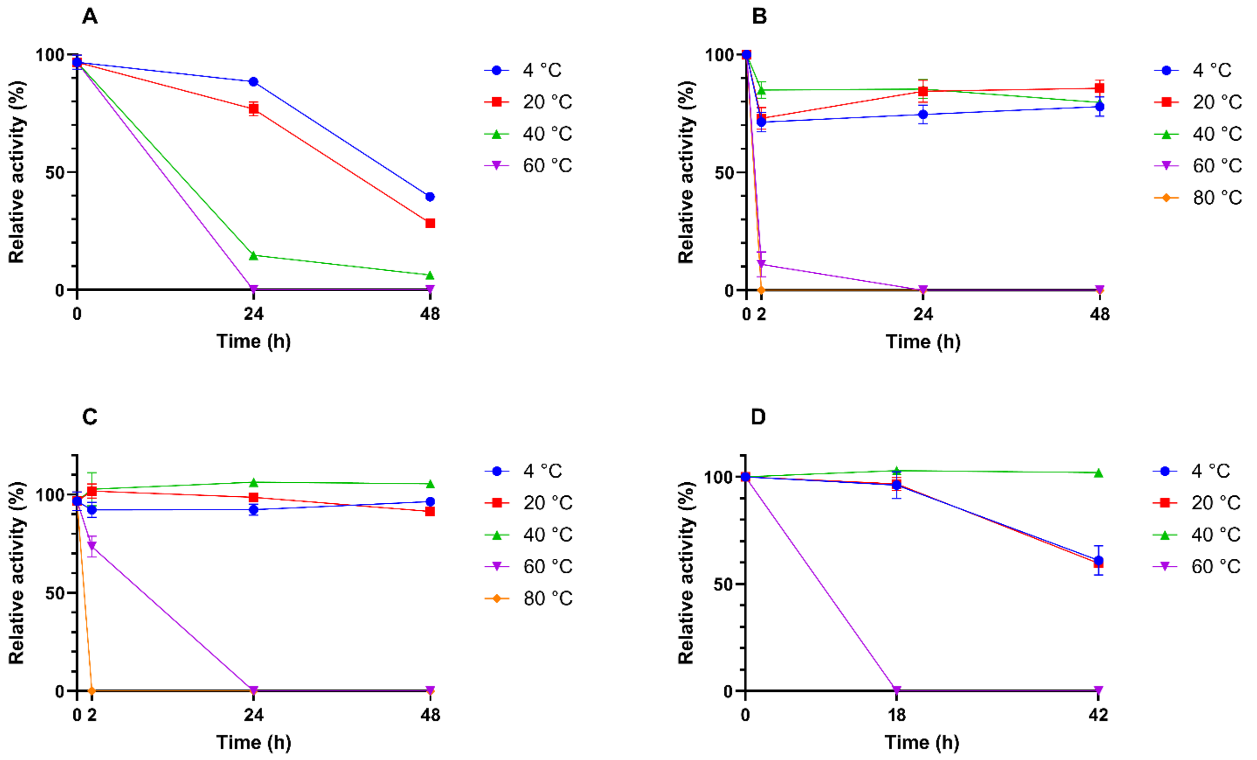

Figure S2. The effect of temperature on the stability of proteases (**A**), esterases (**B**), cellulases (**C**) and xylanases (**D**) produced by the selected strains of *Bacillus* sp.

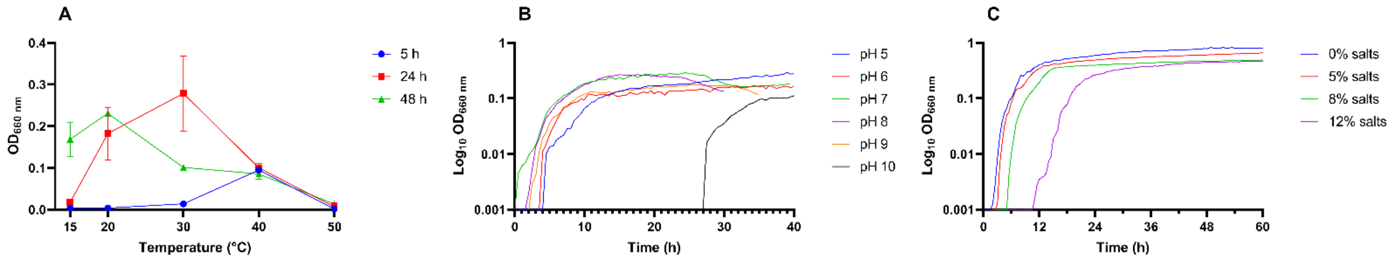

Figure S3. The effect of temperature (**A**), pH (**B**) and salt concentration (**C**) on the growth of *Bacillus* sp. BA N P3.3.

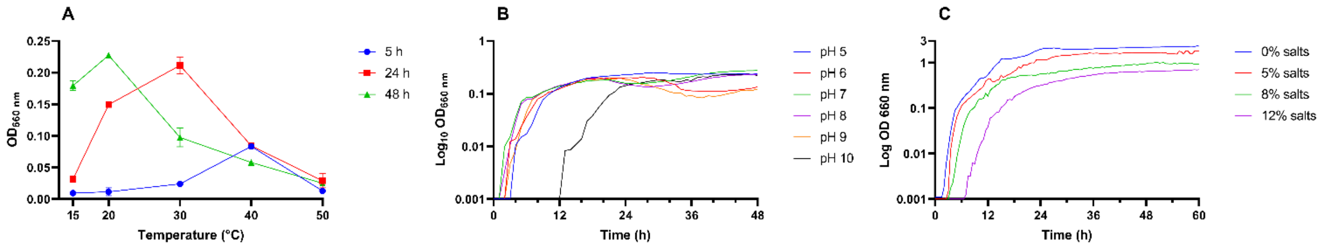

Figure S4. The effect of temperature (**A**), pH (**B**) and salt concentration (**C**) on the growth of *Bacillus* sp. BA N P1.4.

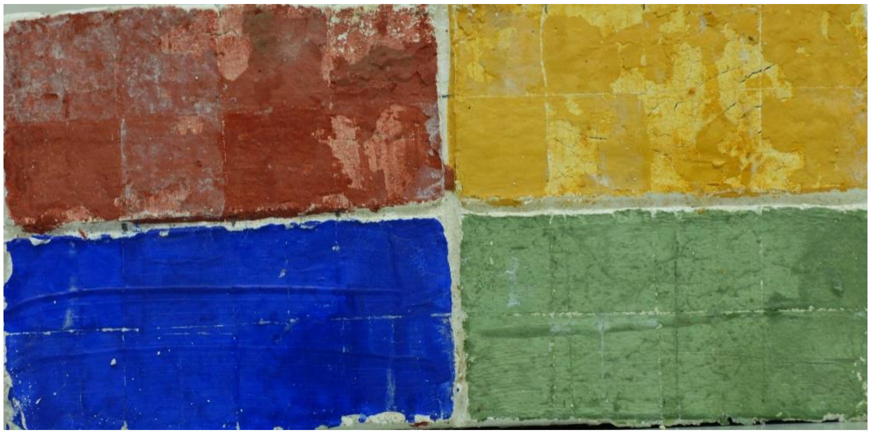

Figure S5. Oil-enriched laboratory model after the treatment with esterase.
